# Supplementary figures and images for: Recognition and reconstruction of cell differentiation patterns with deep learning
Source: PLoS Comput Biol. 2023 Oct 27;19(10):e1011582. doi: 10.1371/journal.pcbi.1011582 (PMC10631711; doi:10.1371/journal.pcbi.1011582)

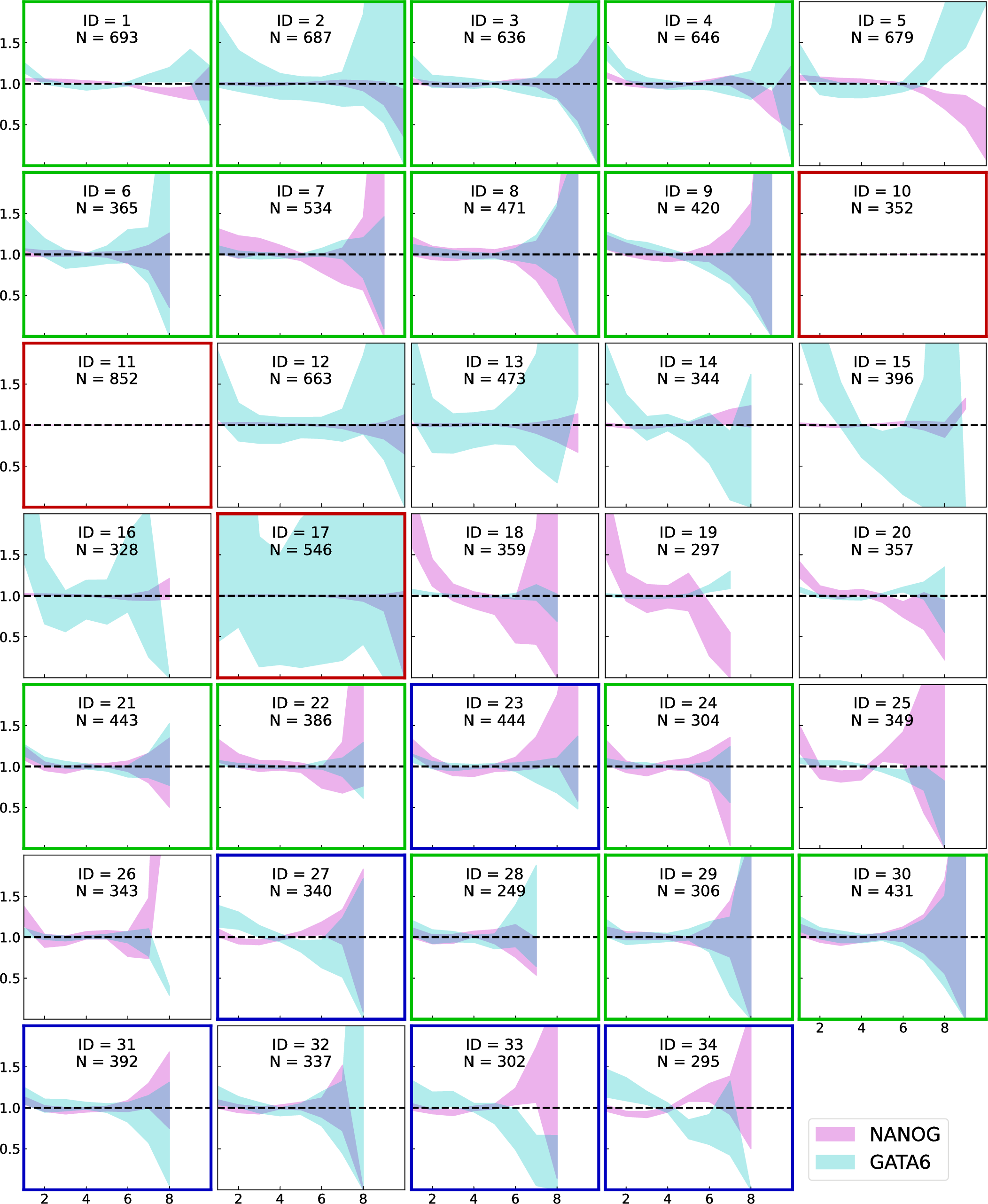

Supplement: S1 Fig — For each envelope, minimum and maximum of 1000 samples were used (see materials and methods for more details). Green borders highlight the organoids that show the most common pattern. Red borders highlight organoids, where cell type proportions are extremely skewed up to the point of no cells of one type. In blue, organoids with a similar pattern, that is not random, are highlighted. (Figure from [33]). (TIF) [file pcbi.1011582.s001.tif]

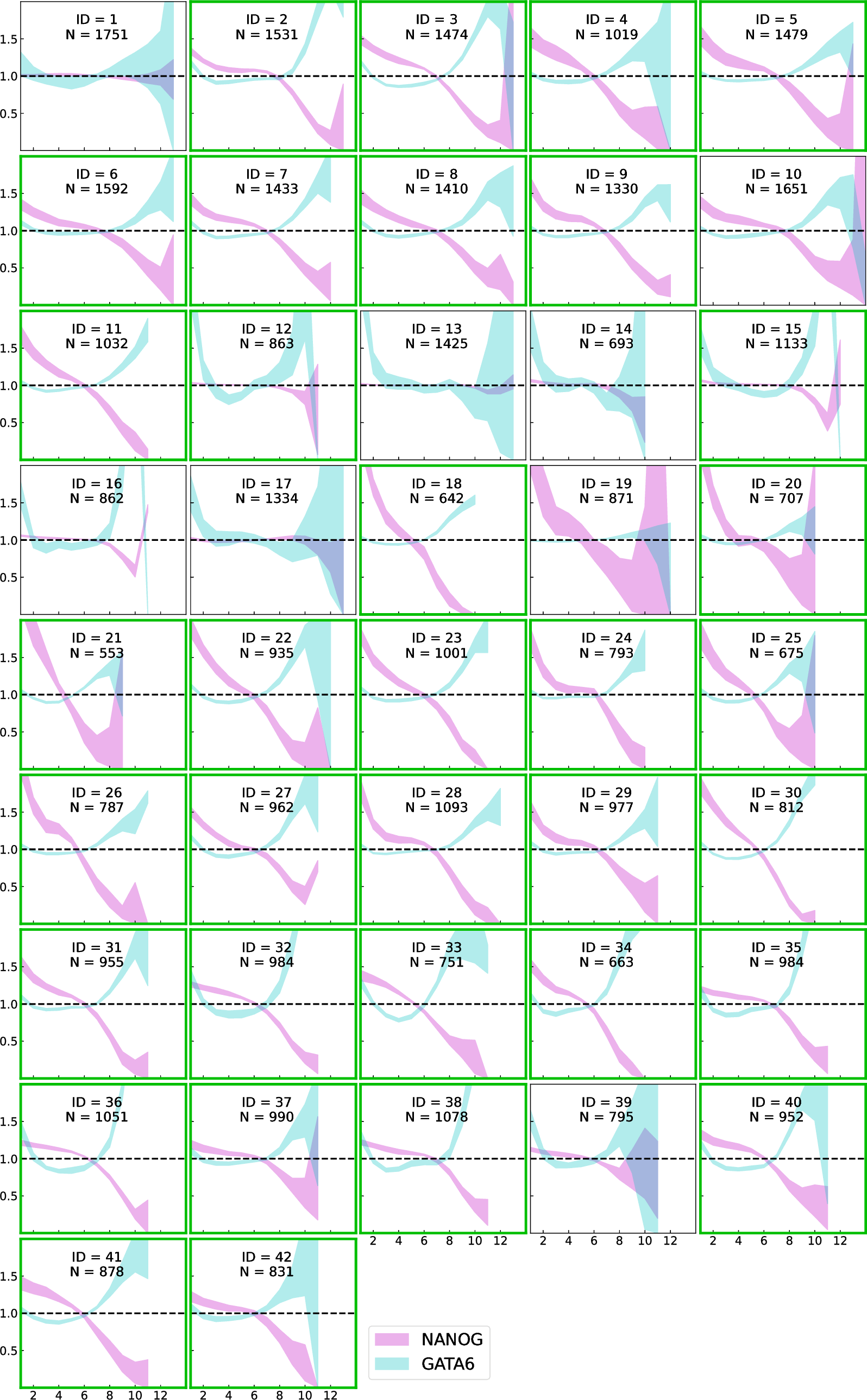

Supplement: S2 Fig — For each envelope, minimum and maximum of 1000 samples were used (see materials and methods for more details). Green borders highlight the organoids that show the most common pattern. (Figure from [33]). (TIF) [file pcbi.1011582.s002.tif]

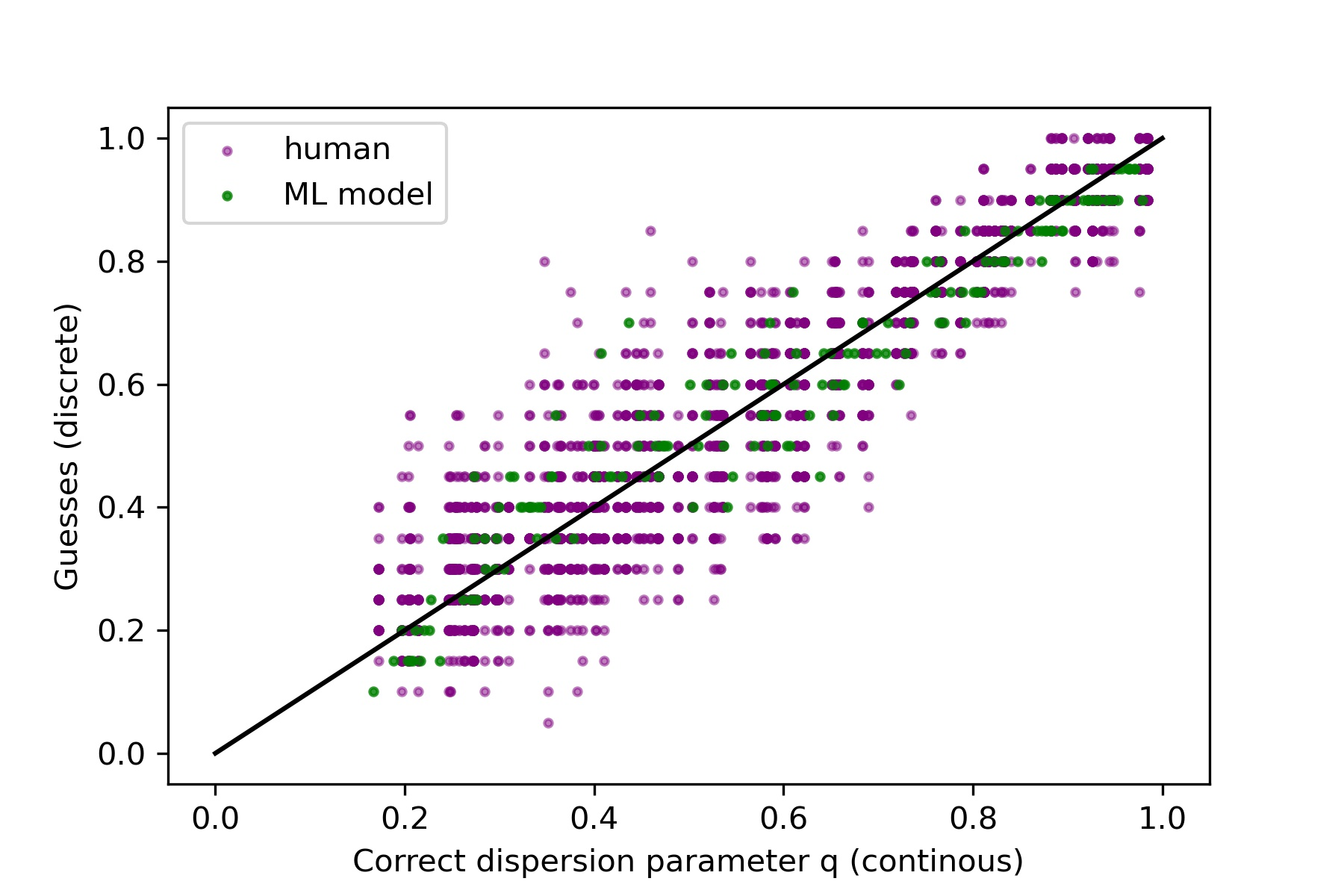

Supplement: S3 Fig — Guesses for the dispersion parameter q versus the correct value for q for Model1.2D and data set A (Table 1). (TIF) [file pcbi.1011582.s003.tif]

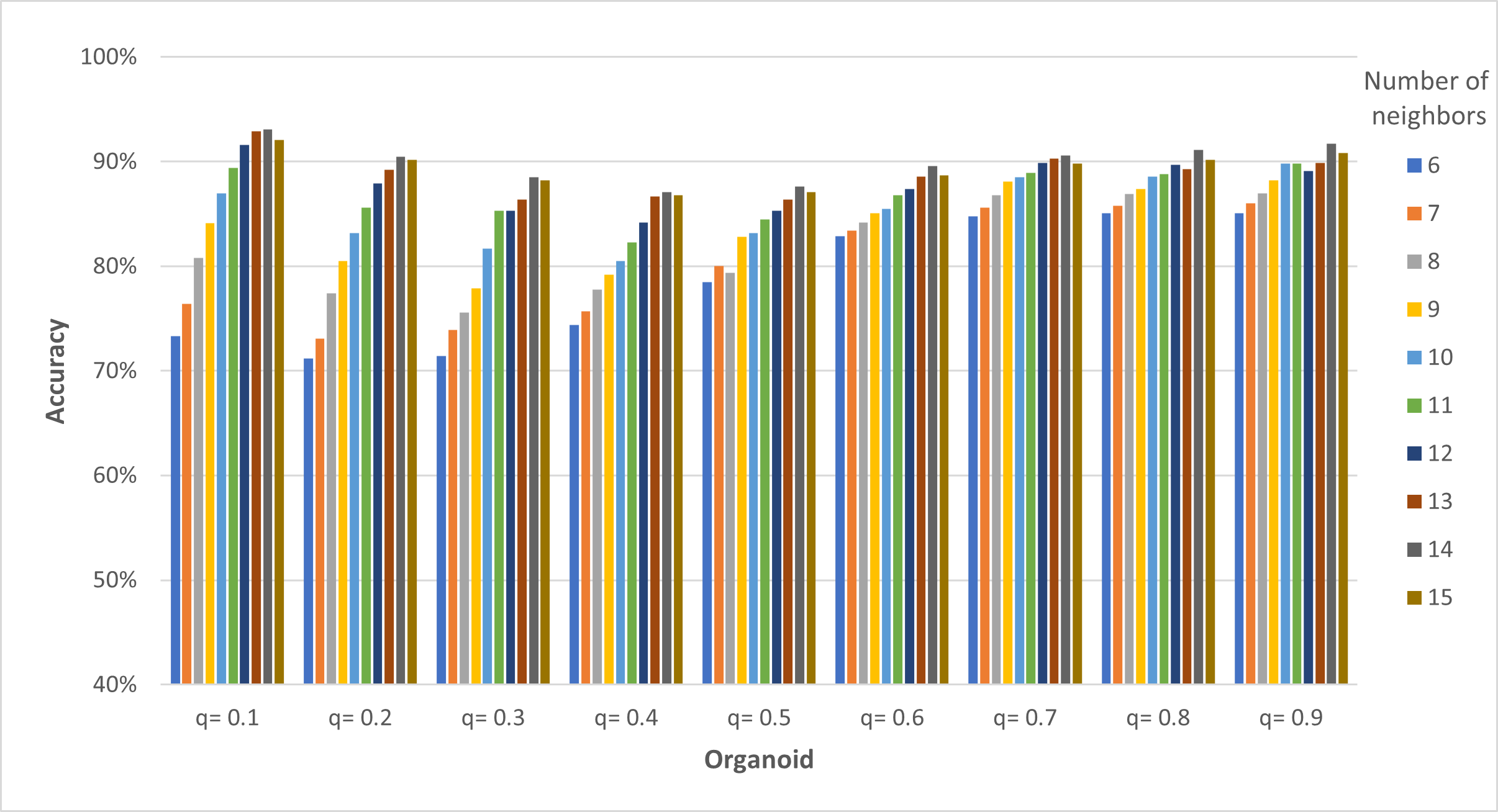

Supplement: S4 Fig — Accuracy of Model2.3D for data set D (Table 1) for different q and different number of neighbors. Please note that for a clearer display, the y-axis starts at 40%. (TIF) [file pcbi.1011582.s004.tif]

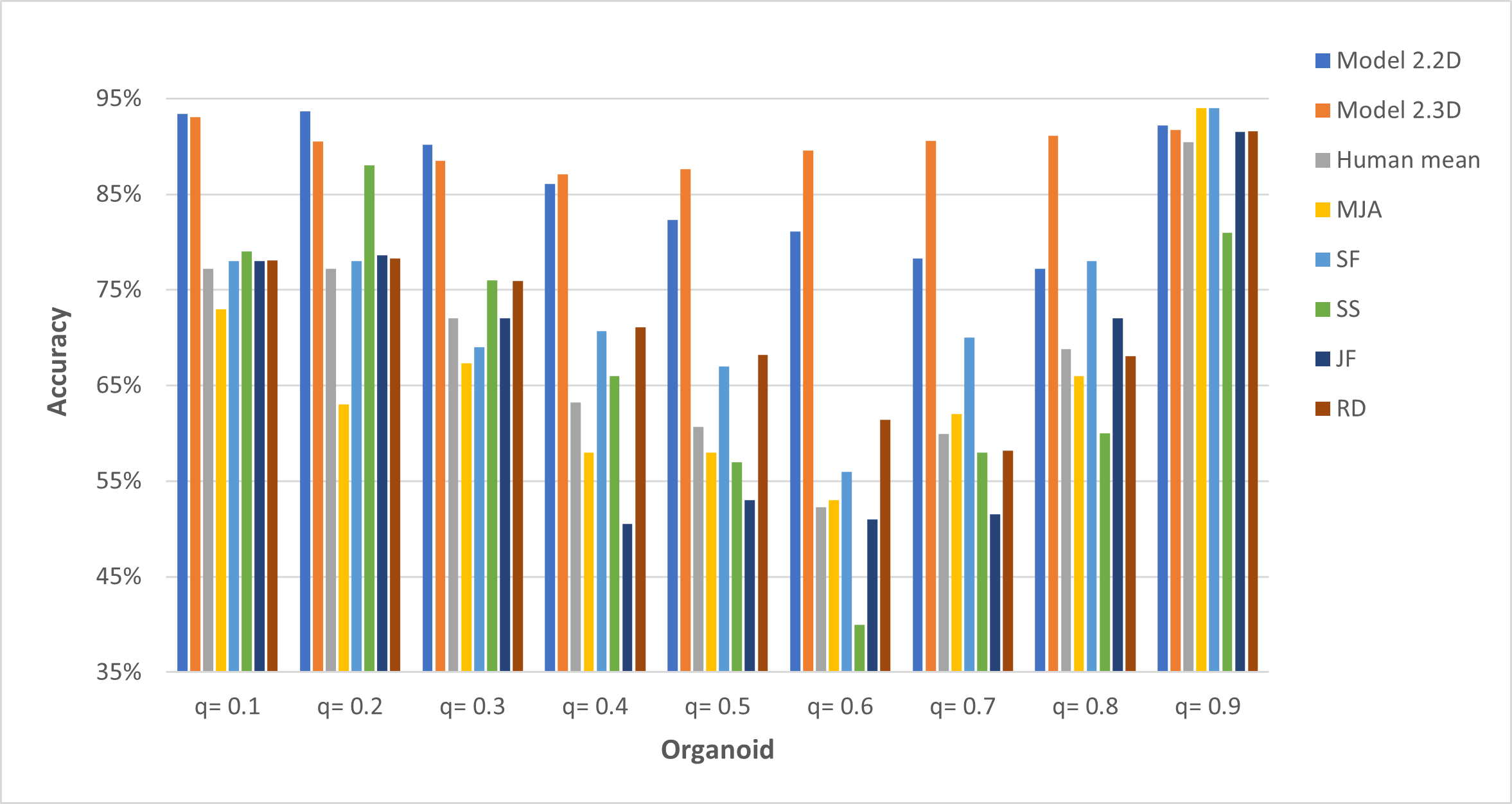

Supplement: S5 Fig — Accuracy of human predictions on simulated data set C (Table 1) by expert as well as the accuracy of Model1.2D and Model1.3D for the different values of q. Please note that for a clearer display, the y-axis starts at 35%. (TIF) [file pcbi.1011582.s005.tif]

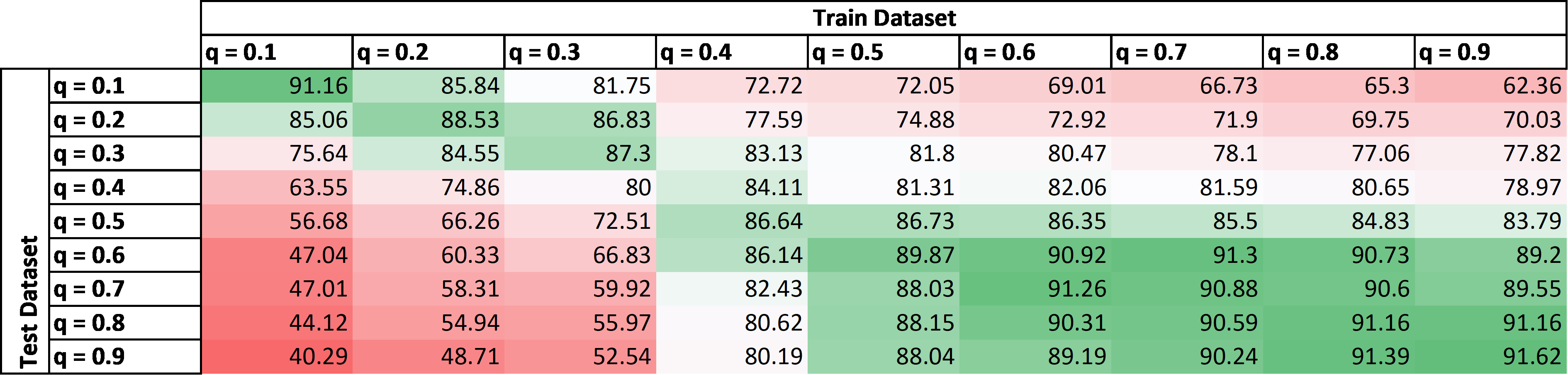

Supplement: S6 Fig — Model2.3D was trained on parts of data set D (Table 1) for the different values of q. Subsequently, these models were used to predict the cell fates in data sets split up by q. Each entry in the matrix corresponds to the accuracy of one such test run. The color coding ranges from dark red (worst accuracy) to dark green (best accuracy). (TIF) [file pcbi.1011582.s006.tif]

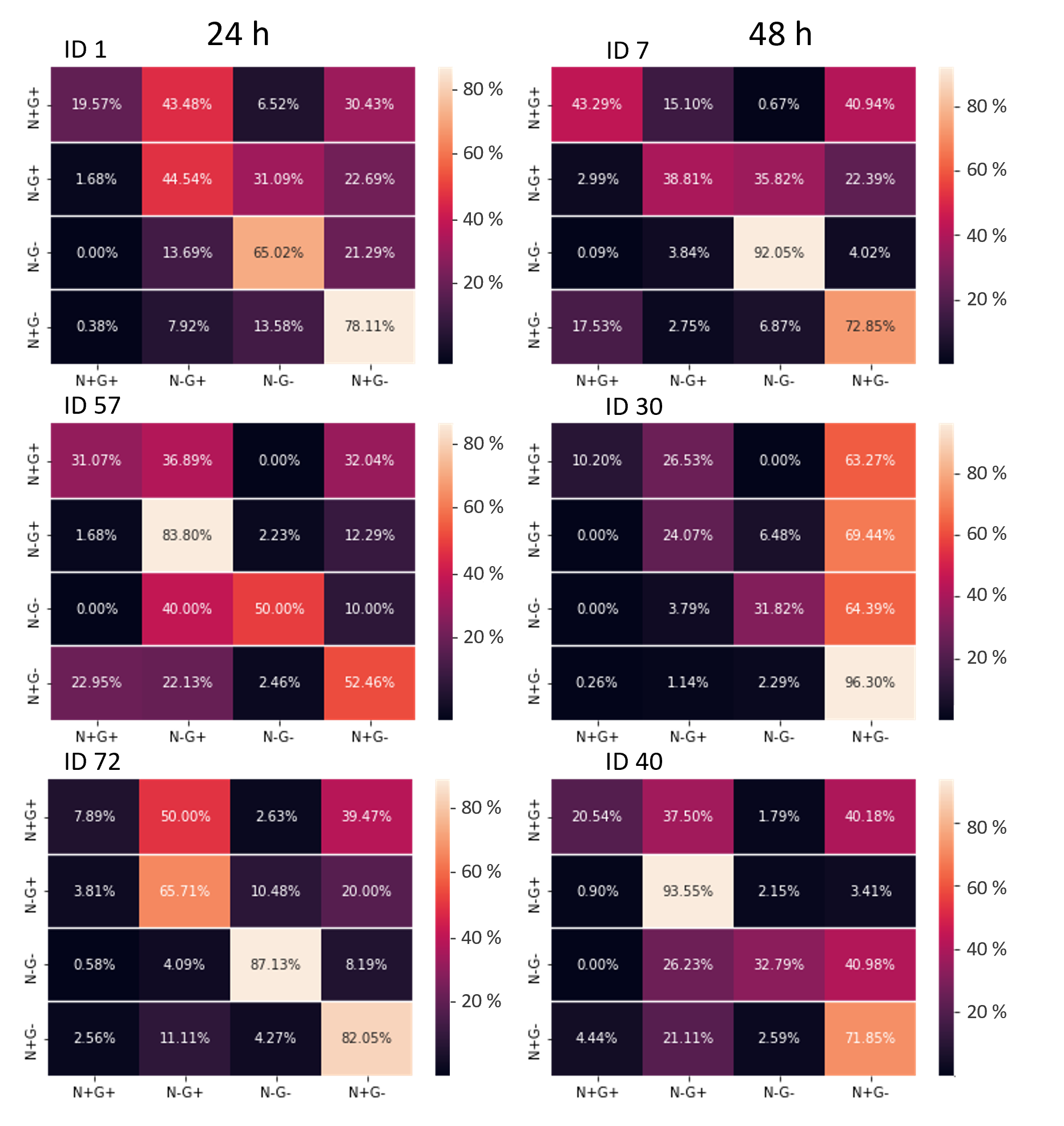

Supplement: S7 Fig — Matrices show predictions plotted against true labels for three 24 h (left) and three 48 h (right) ICM organoids. The rows are normalized to 1. (TIF) [file pcbi.1011582.s007.tif]
