# Supplementary material for: Recognition and reconstruction of cell differentiation patterns with deep learning
Source: PLoS Comput Biol. 2023 Oct 27;19(10):e1011582. doi: 10.1371/journal.pcbi.1011582 (PMC10631711; doi:10.1371/journal.pcbi.1011582)
Supplement: S1 Text — (PDF) [file pcbi.1011582.s008.pdf]

Supplementary material for "Recognition and reconstruction of cell differentiation patterns with deep learning"

Robin Dirk[1◑], Jonas L. Fischer[1◑], Simon Schardt[1◑], Markus J. Ankenbrand[1], Sabine C. Fischer[1*],

1 Julius-Maximilians-Universität Würzburg, Fakultät für Biologie, Center for Computational and Theoretical Biology, Klara-Oppenheimer-Weg 32, Campus Hubland Nord, 97074 Würzburg, Germany

◑These authors contributed equally to this work.
* sabine.fischer@uni-wuerzburg.de

S1 text: Agent-based modeling of cell fate patterns in ICM organoids

Data sets A-D result from simulations in which first an artificial tissue was created and then simulations for transcriptional regulation were performed.

The tissues were created from a single initial cell through cell growth and cell division.

Cell growth Cell growth is implemented by an increase of a cell's radius r such that

$$\frac{dr}{dt} = \lambda(r^* - r), \tag{1}$$

where λ denotes a constant growth rate and r^* the maximum cell radius.

Cell division We used a stochastic process for cell division based on the cell's radius. The cumulative distribution function (CDF) $F(r)$ of a truncated normal distribution defines the probability that a cell will have divided up to some radius r. The CDF is defined via

$$\begin{aligned} &F(r) = \frac{\mathrm{erf}(\rho) - \mathrm{erf}(\rho_{\min})}{\mathrm{erf}(\rho_{\max}) - \mathrm{erf}(\rho_{\min})}, \qquad r \in [r_{\min}, r_{\max}], \\ &\rho = \frac{r - \mu_{\mathrm{div}}}{\sqrt{2}\sigma_{\mathrm{div}}}, \qquad \rho_{\min} = \frac{r_{\min} - \mu_{\mathrm{div}}}{\sqrt{2}\sigma_{\mathrm{div}}}, \qquad \rho_{\max} = \frac{r_{\max} - \mu_{\mathrm{div}}}{\sqrt{2}\sigma_{\mathrm{div}}}, \end{aligned} \tag{2}$$

where μ_{div} and σ_{div} are the mean and standard deviation of the distribution. We chose $\mu_{\mathrm{div}} = \frac{1}{2}(r_{\min} + r_{\max})$ to preserve the symmetry of the distribution despite truncation. The function erf is the error function defined as

$$\mathrm{erf}(x) = \frac{2}{\pi} \int_0^x e^{-x^2}\,\mathrm{d}x. \tag{3}$$

Outside of $[r_{\min}, r_{\max}]$, we define

$$F(r) = 0, \qquad r < r_{\min}, \tag{4}$$

$$F(r) = 1, \qquad r > r_{\max}. \tag{5}$$

Hence, no cell divides up to a radius $r_{\min}$, whereas cells with a radius greater than $r_{\max}$ always divide. In a time discrete process, the division probability cannot be directly calculated from (2).

Instead, previous attempts to divide have to be included as well. The exact division probability of a cell with radius $r^{(N)}$ at time step N is then described by

$$p_i^{(N)} = \frac{F(r_i^{(N)}) - F(r_i^{(N-1)})}{1 - F(r_i^{(N-1)})}. \tag{6}$$

Following cell division, we use mass/volume conservation to calculate the daughter cell's radii r_1 and r_2 as

$$\frac{4}{3}\pi r^3 = \frac{4}{3}\pi r_1^3 + \frac{4}{3}\pi r_2^3. \tag{7}$$

In our model, we assume symmetric cell division, i.e. $r_1 = r_2 =: r_0$. This yields

$$r_0 = \frac{r}{2^{\frac{1}{3}}}. \tag{8}$$

The distance between the daughter cells after division was set to

$$h := |x_1 - x_2| = \frac{r - r_0}{2}(1 + \xi),$$

where ξ describes normally distributed noise with mean 0 and standard deviation 0.1. The orientation of the axis of division was chosen randomly.

Cell-cell adhesion and repulsion The growth of a cell causes the displacement of any adjacent cells. The corresponding equations of motion for n cells were obtained by an overdamped approximation, i.e. inertia was neglected such that the velocities of the cells are proportional to the forces acting on them, i.e.

$$\frac{d\boldsymbol{x_i}}{dt} = F_0 \sum_{\substack{j=1 \\ j \neq i}}^{n} F_{i,j} \frac{\boldsymbol{x}_j - \boldsymbol{x}_i}{|\boldsymbol{x}_j - \boldsymbol{x}_i|}, \qquad \text{for } i = 1, ..., n. \tag{9}$$

Variable $\boldsymbol{x}_i$ describes the centroid of cell i. Parameter F_0 is a constant scaling factor, whereas $F_{i,j}$ describes the magnitude of the force of cell j acting on cell i. The forces are derived from the Morse potential, which has already been successfully applied in similar biological contexts [1, 2]. They can be written in terms of cell radii r_i, r_j and positions $\boldsymbol{x}_i$, $\boldsymbol{x}_j$ such that

$$F_{i,j} = \begin{cases} 2\alpha \left(e^{-\alpha(|\boldsymbol{x}_j - \boldsymbol{x}_i| - \sigma(r_j + r_i))} - e^{-2\alpha(|\boldsymbol{x}_j - \boldsymbol{x}_i| - \sigma(r_j + r_i))} \right) & \text{for } |\boldsymbol{x}_j - \boldsymbol{x}_i| \leq r_j + r_i, \\ 0 & \text{for } |\boldsymbol{x}_j - \boldsymbol{x}_i| > r_j + r_i. \end{cases} \tag{10}$$

The parameter α describes the stiffness of the cells, whereas $\sigma \in (0, 1]$ defines the optimal distance between two cells in contact as a fraction of the sum of their radii. The effect of the forces depends on the relative position of two cells. If the cells are too close, they will repel, whereas they will adhere to each other if they are further apart but still in contact. In between, an optimal state will be found. If the distance between the cells exceeds the sum of their radii, there can be no physical interaction.

For the different data sets, this procedure of cell growth and cell division was performed for different numbers of time steps to reach the intended tissue sizes. Subsequently, the tissues were kept fixed and the transcriptional regulation was performed on the fixed tissues.

Transcriptional regulation We employed our previously developed model for transcriptional regulation [3]. It follows the temporal evolution of two transcription factors u and v based on a model derived from statistical thermodynamics. The corresponding gene regulatory network (GRN) involves mutual inhibition of u and v as well as their auto-activation. An external signal s is used to inhibit u while also activating v. For a tissue with N cells, we end up with a system of ordinary differential equations

$$\begin{aligned}\frac{du_i}{dt} &= r_u \frac{\eta_u u_i}{1 + \eta_v v_i (1 + \eta_s \eta_{vs} s_i) + \eta_u u_i + \eta_s s_i} - \gamma_u u_i \\ \frac{dv_i}{dt} &= r_v \frac{\eta_v v_i (1 + \eta_s \eta_{vs} s_i)}{1 + \eta_v v_i (1 + \eta_s \eta_{vs} s_i) + \eta_u u_i + \eta_s s_i} - \gamma_v v_i, \qquad i = 1, ..., N,\end{aligned} \tag{11}$$

with the energy coefficients $\eta_x = e^{-\Delta\epsilon_x}$, the transcription rates r_x and the decay rates γ_x.

We consider a distance-based neighbour signal s that is given as the result of the expressions of u in any other cell according to

$$s_i = \left(\sum_{j \neq i} u_j q^{d_{ij}-1} \right) \Big/ \left(\max_k \sum_{j \neq k} q^{d_{kj}-1} \right), \qquad q \in [0, 1]. \tag{12}$$

Here, d_{ij} denotes the distance of cells i and j in the cell graph. By this definition of s_i, the parameter q represents the strength of signal dispersion throughout the tissue. A value of $q = 0$ means that only direct neighbors influence a cell's fate. Increasing q increases the influence of cells further away.

Table S I: List of model parameters and initial conditions together with their values and descriptions.

Model parameter	Fixed value	Description
T_{grow}	$\in \{25, 30, T_{24}, T_{48}\}$	Colony/Organoid growth time
T_{trans}	100	Time for transcriptional regulation
T_{24}	23.09	Time to reach same average as 24h Organoids (441.73 cells)
T_{48}	27.06	Time to reach same average as 48h Organoids (1041.24 cells)
N_{grow}	6000	Number of time steps for colony/organoid growth
N_{trans}	1000	Number of time steps for transcriptional regulation
r^*	1	Maximum radius
λ	0.5	Cell growth rate
h_i	$\frac{(1-2^{-1/3})}{2} r_i (1+\rho)$	Distance of daughter cells after division (r_i = mother cell radius)
ρ	$\rho \sim N(0, 0.1)$	Random noise for cell division distance
F_0	0.01	Displacement force scaling factor
α	3	Cell stiffness
σ	0.7	Cell-cell distance optimality factor
$-\Delta\varepsilon_u$	$\in (6, 7.87)$	Energy difference w.r.t. binding of u
$-\Delta\varepsilon_v$	6	Energy difference w.r.t. binding of v
$-\Delta\varepsilon_s$	2	Energy difference w.r.t. binding of s
$-\Delta\varepsilon_u$	2	Energy difference w.r.t. combined binding of v and s
r_u	1	Transcription rate of u
r_u	1	Transcription rate of v
r_v	10	Decay rate of u
r_v	10	Decay rate of v

References

[1] Middleton AM, Fleck C, Grima R. A continuum approximation to an off-lattice individual-cell based model of cell migration and adhesion. Journal of Theoretical Biology. 2014;359:220 – 232. doi:https://doi.org/10.1016/j.jtbi.2014.06.011.

[2] Stichel D, Middleton AM, Müller BF, Depner S, Klingmüller U, Breuhahn K, et al. An individual-based model for collective cancer cell migration explains speed dynamics and phenotype variability in response to growth factors. npj Systems Biology and Applications. 2017;3(1):5. doi:10.1038/s41540-017-0006-3.

[3] Schardt S, Fischer SC. Adjusting the range of cell–cell communication enables fine-tuning of cell fate patterns from checkerboard to engulfing. Journal of Mathematical Biology. 2023;87(4):54. doi:10.1007/s00285-023-01959-9.
