# Supplementary material for: Recognition and reconstruction of cell differentiation patterns with deep learning
Source: PLoS Comput Biol. 2023 Oct 27;19(10):e1011582. doi: 10.1371/journal.pcbi.1011582 (PMC10631711; doi:10.1371/journal.pcbi.1011582)
Supplement: S2 Text — (PDF) [file pcbi.1011582.s009.pdf]

Supplementary material for "Recognition and reconstruction of cell differentiation patterns with deep learning"

Robin Dirk[1◑], Jonas L. Fischer[1◑], Simon Schardt[1◑], Markus J. Ankenbrand[1], Sabine C. Fischer[1*],

1 Julius-Maximilians-Universität Würzburg, Fakultät für Biologie, Center for Computational and Theoretical Biology, Klara-Oppenheimer-Weg 32, Campus Hubland Nord, 97074 Würzburg, Germany

◑These authors contributed equally to this work.
* sabine.fischer@uni-wuerzburg.de

S2 text: Pair correlation function

Calculating the pair correlation functions (PCFs) for patterns of two cell types A and B requires counting different types of cell pairings for certain distances. In order to describe the PCF mathematically, we first introduce various sets. The set S_k describes all the cell pairs (i, j) found at a distance d_{ij}. Similarly, S_k^A denotes the set of all A cell pairs with distance k. Analogously, S_k^B is defined for B cell pairs. Finally, the two sets T^A and T^B contain all cells A and cells B, respectively. In mathematical notation, we write

$$S_k = \left\{(i,j) \in \mathbb{N}^2 : d_{ij} = k,\ 1 \le i,j \le N\right\}, \tag{1}$$

$$S_k^A = \{(i,j) \in S_k : i \text{ and } j \text{ are } A \text{ cells}\}, \tag{2}$$

$$S_k^B = \{(i,j) \in S_k : i \text{ and } j \text{ are } B \text{ cells}\}, \tag{3}$$

$$T^A = \{i \in \mathbb{N} : i \text{ is } A \text{ cell}\, 1 \le i \le N\}, \tag{4}$$

$$T^B = \{i \in \mathbb{N} : i \text{ is } B \text{ cell}\, 1 \le i \le N\}. \tag{5}$$

In the next step, we want to find the proportions of the number of equal pairings by looking at the ratio of cell pairs of equal type and pair of any type. We denote the cardinality, i.e. the number of elements, of a set S by $|S|$. Thus, we calculate the proportions $r_{AA}(k)$ of A pairs at distance k as well as the proportions $r_{BB}(k)$ of B pairs at distance k via

$$r_{AA}(k) = \frac{|S_k^A|}{|S_k|} \quad \text{and} \quad r_{BB}(k) = \frac{|S_k^B|}{|S_k|}. \tag{6}$$

The goal is to relate these proportions with the probability of randomly picking two cells of equal type. For this, we need the total number of A cells T^A and B cells T^B. The chance of picking one A cell is $|T^A|$. If one has already been picked, then the remaining chance of picking a second one becomes $(|T^A| - 1)/(N - 1)$. In total, we can write the probability of randomly selecting two A cells or two B cells as

$$p_{AA} = \frac{|T^A|(|T^A| - 1)}{N(N-1)} \quad \text{and} \quad p_{BB} = \frac{|T^B|(|T^B| - 1)}{N(N-1)}. \tag{7}$$

Combined, the PCFs measure the ratios of A or B cell pairs within every possible distance normalized by the probability of finding these cell pairs, i.e.

$$\rho_A(k) = \frac{r_{AA}(k)}{p_{AA}} = \frac{|S_k^A|N(N-1)}{|S_k||T^A|(|T^A|-1)}, \tag{8}$$

$$\rho_B(k) = \frac{r_{BB}(k)}{p_{BB}} = \frac{|S_k^B|N(N-1)}{|S_k||T^B|(|T^B|-1)}. \tag{9}$$

For a uniformly distributed amount of A or B cells, the correlation function returns a value close to 1 for every cell distance k. Consequently, deviations from 1 yield information about how much more or fewer equal cell pairs are found in certain ranges.
