# Supplementary material for: Recognition and reconstruction of cell differentiation patterns with deep learning
Source: PLoS Comput Biol. 2023 Oct 27;19(10):e1011582. doi: 10.1371/journal.pcbi.1011582 (PMC10631711; doi:10.1371/journal.pcbi.1011582)
Supplement: Attachment — Submitted filename: ResponsesReviewers.pdf [file pcbi.1011582.s010.pdf]

Responses to reviewer comments for "Recognition and reconstruction of cell differentiation patterns with deep learning"

Dear Jinyan Li and Daniel Beard,

thank you very much for assessing our work. To address the reviewer comments we have made some major additions to our manuscript. We added results based on spatial statistics together with the corresponding methods and substantially reworked the discussion. Furthermore, we added the details of the mathematical model as a supplementary text. Please find the detailed responses to the reviewers comments below in italics.

Sincerely,
Sabine Fischer

Reviewer #1: The manuscript submission by Dirk et al describes a series of machine learning algorithms designed to investigate the characteristics associated with inner cell mass (ICM) organoids, which capture an early stage of blastocyst morphogenesis in cultured murine embryonic stem cells. The authors generate a large synthetic dataset of 2D and 3D cell configurations that describe binary state transitions of differentiation according to a "dispersion" parameter that accounts for influences on a cell ranging from only nearest neighbors to influences from cells further away and yields striking differences in multicellular patterning of the system. By generating data from thousands of organoids, a graphical neural network (GNN) model was trained and validated to predict the dispersion parameter value for a given image. The GNN predicts for actual experimental organoid images a range of dispersion values. Another ML model generates predictions of a cell phenotype (one of 4 classes based upon Nanog/Gata6 expression) solely based upon information regarding the closest neighbors' phenotypes. While the model performs excellent for the synthetic dataset, accuracy of the experimental images is good but with considerable room for improvement. Primary claims in the paper are 1) algorithms can detect patterns that humans can't; and 2) cell fate reconstruction can be predicted based upon a subset of the nearest neighbors.

The rigor and implementation of the machine learning in this paper is quite good. The use of a computational model yielded insights into the perception of cellular patterning detectable by ML versus human.
We thank the reviewer for the appreciation and positive evaulation of our work and the constructive comments below.

However in the paper there are several critical shortcomings.

MAJOR:

1. Throughout the paper, the authors make claims (including the title) that the prediction of an individual cell's phenotype from the neighboring cell information is pattern reconstruction. I find this misleading, one can think of this more as "spatial imputing" in which enough information about the surroundings allows for an estimate of what is happening at the center cell. That is quite different than pattern prediction which occurs on the macroscale.

We thank the reviewer for this comment as it helps to make our work accessible to a wider audience. A careful check of the literature suggested to us that the boundaries between pattern reconstruction and spatial imputation are not entirely clear. Therefore, we decided to refer to our approach by "pattern reconstruction through spatial imputation" and included this in the introduction, the methods, the results and the discussion part.

As a sidenote, the motivation for the entire exercise is a bit weak. Why do we want to predict a single cell's fate? Pivoting the emphasis toward imputation for spatial transcriptomics data acquisition could be an improvement to the paper.
This is a very interesting thought. We would like to keep the focus of the paper on the biological question of the interactions in the ICM organoids, but we have pointed out the potential value of our approach for spatial transcriptomics at the end of the discussion.

2. The only description of the model is a reference to a pre-print that is not peer-reviewed. The authors need to provide enough description so that the readers can understand the nature of the model. Is it agent-based? Does the model contain any stochasticity or will the same initialized set yield the same outcome? Why is there variability in the organoid cell numbers for dataset B? How does the reader interpret the meaning of model-defined or experimentally extracted dispersion values?

The mentioned pre-print has now been accepted for publication in the Journal of Mathematical Biology and we are currently waiting for the proofs. Hence, at the next stage, we should be able to include the peer-reviewed reference. Nonetheless, we have included a detailed description of the model together with the parameter values used as a supplementary text. The reasons for the variability in cell number in the different data sets were included in the Materials and Methods section.

3. Similarly, the authors never show any examples of the organoid images that they are working with. Statements in the paper such as "the spatial arrangement of epiblast and primitive endoderm precursor cells is non-random but visually unrecognizable" should be backed up by these images. The authors should show an example of where inaccurate predictions occur spatially on the organoid.

We included example microscopy images as Figure 6. Examples of accurate and inaccurate predictions are provided as interactive visualisations here: https://rodirk.github.io/patternReconstruction/ (https://rodirk.github.io/patternReconstruction/) and

referenced in the main text.

4. The estimation of cell fate by both machine and expert is made from cell state and rank order distance from the predicted cell. All spatial information is lost and I don't understand the choice in discarding this. For example, in Figure 5, if the user knew that all the red cells were clustered to the left side of the cell in question, and the blue cells were clustered to the right, that information has both biological implications (e.g. a larger localized buildup of morphogen) and implications for accuracy.

Our current approach is based on the biological hypothesis that the cells communicate via a spatially unbiased signaling mechanism. But it would be interesting to include the spatial distribution in future studies. We have added respective explanations to the results and the discussion section.

5. The authors' choice in binning their cell data does not normalize expression levels by cell area, and subsequent analysis is based upon the centroid coordinates. How are the results impacted if normalization is performed, which will change the distribution of N-G-, N+G-, N+G+, N-G+ cells?

The underlying experimental data of our study consists of fluorescence images with cell nuclei labelling. Hence, unfortunately, we do not have the cell area available. Our expression levels however are averages per cell nucleus and hence normalised by cell nucleus size, which we have to take as a cell size approximation.We have added this information in the methods subsection "experimental data".

6. Why do bigger, older organoids have higher dispersion values? This result is not sufficiently discussed.

We added a section on summary statistics and expanded the discussion. With these new parts it should be clearer what higher dispersion values would mean for the cell fate patterns.

7. I do not understand the conclusion drawn by the sentences lines 444-447: "For the ICM organoid data, we obtained an accuracy of more than 70% for nine neighbors, which is less than the typical 14 cells that are directly in contact with a given cell. This is in agreement with a short range cell-cell communication as predicted by the pattern recognition".

We thank you for this comment. To avoid misunderstandings and in light of our newly added results for spatial statistics, we have removed this sentence.

MINOR:
8) Throughout the text are references to "2D organoids". These are 2D colonies.

Thank you for pointing this out. We changed the wording accordingly.
10) The justification for discarding organoids with > 2/3 of one type is weak. How many organoids fell into this category? The radially organized organoids that appear at this stage

may have a central core of less than 1/3 and highly relevant to the morphogenesis under investigation
The motivation for the threshold of 2/3 was based on the experimental findings that in the mouse embryo, there are 2/3 primitive embryo vs 1/3 epiblast cells. We have included this informtaion together with the number of discarded organoids.

Reviewer #2: Dirk et al combine machine learning (ML) algorithms and mathematical models to analyze spatial patterns of cellular fate decisions. More specifically, they assume a mathematical model of cellular fate decision where cells can interact with their neighbors and adopt two different fates. In this model, a parameter, q, sets the range of cell-cell interaction. Then, they train ML algorithms on patterns of cell fate decision generated in silico via their model to predict the parameter q, given a specific spatial distribution of cell fates (what they call "pattern recognition" task). Additionally, they train ML algorithms to predict the fate of a cell from the fates of the neighboring cells ("pattern reconstruction" task).
In doing so, they use simulated and imaging data generated from mouse ICM organoids. When applying their model to mouse ICM organoids data, they conclude that the analysis suggests the existence of a short-range cell-cell communication controlling cellular fate decision, in agreement with some experimental evidence.

The approach used by the authors (the combination of ML with mathematical modelling) is very interesting, and very much needed in biology, where there's an increasing availability of large-scale datasets from which we can extract potentially interesting patterns. However, we're still largely unable to obtain valuable insights into the molecular interactions that generate them. The combination of approaches from ML and mathematical modelling can help address this issue.
Overall the implementation of the models is sound, and the manuscript is well-written.
We thank the reviewer for the appreciation and positive evaulation of our work and the constructive suggestions for improvements below.

However, I have some points that should be addressed before publication.

1. Can one predict the value of q (in both 2D and 3D) by computing some spatial statistics used in marked point processes, such as the Stoyan's mark correlation function, the mean or variance-mark function, etc.? Before using more complex approaches based on deep learning, The authors should show that more straightforward strategies like this do not work or are less accurate. This would be more compelling than comparing the accuracy of the deep learning algorithms with the manual annotation of human experts.

We included Moran's index and pair correlation functions for the experimental data and showed the results for matching experimental data based on these measurements exemplarily for six organoids. This is included as a new paragraph in the results section. The respective methods can be found in the methods section and we included this aspect in the introduction and the discussion.

2. For some values of q, the authors observed a decrease in the model performance in the task of pattern reconstruction. For example, in 2D using Model 2, the testing accuracy was below 80% for higher values of q (line 355), where engulfing patterns are obtained. Does the performance improve if, in addition to the cell fate and distances of the nearest neighbors, the cell's position is also given as input (e.g., using some sort of distance from the "boundary")? It seems like, especially in some situations (like in the engulfed patterns), the relative position of the cell might also be predictive of its fate. It would be interesting to see whether the addition of the cell position can improve the models' accuracy with the in vitro data.

We tested including the coordinates for x,y,z as cartesian as well as radial coordinates in the model and got a decrease in accuracy. We have added this to the results section.

3. Given that 4 categories are used with the in vitro data, the authors should generate in silico data with 4 categories as well (instead of 2) and then compare the accuracy they obtain with the in silico vs the in vitro data. This would also be useful to support their claim that the lower accuracy observed with the in vitro data is (partly) due to the greater number of categories.

Unfortunately, generating simulated data with four categories is not straight forward. Our modelling approach can at most result in three steady states: u+v- and v+u- as well as u+v+ or u-v- (Schardt, PhD thesis). It would be interesting to extend our model to generate four states in the future. We included this aspect in the discussion.

Have the authors made all data and (if applicable) computational code underlying the findings in their manuscript fully available?
The PLOS Data policy requires authors to make all data and code underlying the findings described in their manuscript fully available without restriction, with rare exception (please refer to the Data Availability Statement in the manuscript PDF file). The data and code should be provided as part of the manuscript or its supporting information, or deposited to a public repository. For example, in addition to summary statistics, the data points behind means, medians and variance measures should be available. If there are restrictions on publicly sharing data or code —e.g. participant privacy or use of data from a third party— those must be specified.
Reviewer #1: Yes
Reviewer #2: None

PLOS authors have the option to publish the peer review history of their article (what does this mean?). If published, this will include your full peer review and any attached files.

If you choose "no", your identity will remain anonymous but your review may still be made public.

Do you want your identity to be public for this peer review? For information about this choice, including consent withdrawal, please see our Privacy Policy.
Reviewer #1: No
Reviewer #2: No
