# Supplementary material for: Recognition and reconstruction of cell differentiation patterns with deep learning
Source: PLoS Comput Biol. 2023 Oct 27;19(10):e1011582. doi: 10.1371/journal.pcbi.1011582 (PMC10631711; doi:10.1371/journal.pcbi.1011582)
Supplement: Attachment — Submitted filename: 2023_09_08_ReviewerResponses.pdf [file pcbi.1011582.s011.pdf]

Dear Jinyan Li and Daniel Beard,

thank you very much for the additional comments. We have adjusted the manuscript accordingly and provide detailed responses to the reviewers comments below in italics.

Sincerly,
Sabine Fischer

Reviewer #1: The authors have done an excellent job revising the manuscript to address concerns. The addition of spatial autocorrelation metrics greatly enhances the paper. I found the interactive web visualization very useful for exploring the data. I do have lingering comments that were not fully addressed by the edits:

1. The referencing of 2D systems as organoids still appears throughout the document, e.g. Figure 2, lines 229, 368
   We thank the reviewer and apologise for this inconsistency. We carefully checked all appearances of the term "organoids" and should now have fully adressed this comment.

2. Lines 323-325: "We find that 24 h after organoid formation, the Moran's index is consistently larger than 0, indicating a non-random distribution of the cells (Fig. 7). For 48 h organoids, the average Moran's index increases further, indicating stronger clustering of the two cell types."
   It is unclear which dataset is being referred to here. It would be helpful to explicitly label the dataset used for this observation. Is it experimental images or simulations?
   Thank you for pointing out this ambiguity. These results refer to data set E. We have indicated this in the text and the corresponding figure.

3. Unfortunately, despite the addition of Figures 8, 9, 10, S1, S2, there is an unclear degree of overlap in results from other publication. Whatever has been accepted for publication must be quite different from the arxiv link. How much of what is being presented is new analyses on data presented in the other paper versus reproductions of the same figures from that paper? Typically, with a large degree of overlap, a journal will request the accompanying pre-print to make this determination.
   Thank you for pointing this out. We think that several points have led to this comment and will try to clarify these here:
   The modelling paper has now been published and we have adjusted the reference accrodingly: *https://link.springer.com/article/10.1007/s00285-023-01959-9 It contains the development and analysis of the transcriptional part of the mathematical model described in Supplementary text 1. It does not include any analysis on the experimental ICM organoid data.*
   The references for Figs 8, 9, 10, S1, S2 were wrong. They were not from the modelling paper but from the PhD thesis of Simon Schardt: https://opus.bibliothek.uni-wuerzburg.de/frontdoor/index/index/docId/30194 We have adjusted this accordingly.
   The only further overlaps that occur are with the arXiv link: https://arxiv.org/abs/2212.10058
   Hence, there are no overlapping figures with published papers or papers accepted for publication.

4. I find figure 10 very hard to interpret. I understand that the attempt is to show "good" versus "bad" organoids modeled at different q values, however the use of opacity in the image does not make sense to me. Am I to interpret this as only organoid 36 is well-captured by the Moran's index statistic? What are the implications of this result?
   Thank you for pointing out this source of misunderstanding. We have expanded the main text and the figure legend to make the purpose and message of the figure clearer.
   Reviewer #2: The authors have addressed all the points that I've raised, and I have no further comments.
